# Supplementary material for: The Effect of Silver Nanoparticle Addition on Micropropagation of Apricot Cultivars (Prunus armeniaca L.) in Semisolid and Liquid Media
Source: Plants (Basel). 2023 Apr 3;12(7):1547. doi: 10.3390/plants12071547 (PMC10097347; doi:10.3390/plants12071547)
Supplement: Supplementary file 1 [file plants-12-01547-s001.zip › plants-2315012-supplementary.pdf]

Table S1. Polynomial equations calculated for predicted values of variables in which significant differences between treatments for each cultivar were found. N.D.= no differences were found.

**Variables**	**Polynomial equations**
**Silver content**	
SSM ‘Canino’	y= 2.625+0.036 x
SSM ‘Mirlo Rojo’	y= 1.124+0.088 x-0.0006 x^2
TIS ‘Canino’	y= 2.384+1.034 x-0.0076 x^2
TIS ‘Mirlo Rojo’	y= 3.615+0.727 x-0.0050 x^2
**Proliferation**	
SSM ‘Canino’	y= 2.100-0.009 x
SSM ‘Mirlo Rojo’	y= 1.480-0.004 x
TIS ‘Canino’	y= 2.410+0.038 x
TIS ‘Mirlo Rojo’	y= 1.100+0.008 x
**Shoot length**	
SSM ‘Canino’	N.D.
SSM ‘Mirlo Rojo’	N.D.
TIS ‘Canino’	y= 1.904-0.014 x-0.0001 x^2
TIS ‘Mirlo Rojo’	N.D.
**Productivity**	
SSM ‘Canino’	y= 2.900-0.012 x
SSM ‘Mirlo Rojo’	y= 1.810-0.005 x
TIS ‘Canino’	y= 4.130+0.048 x
TIS ‘Mirlo Rojo’	y= 1.370+0.013 x
**Leaf surface**	
SSM ‘Canino’	N.D.
SSM ‘Mirlo Rojo’	y= 0.905+0.010 x-0.0004 x^2+0.000004 x^3
TIS ‘Canino’	N.D.
TIS ‘Mirlo Rojo’	N.D.
**Fresh weight**	
SSM ‘Canino’	y= 380.600-2.600 x
SSM ‘Mirlo Rojo’	N.D.
TIS ‘Canino’	y= 867.440+9.370 x
TIS ‘Mirlo Rojo’	y= 467.400+8.600 x
**Dry weight**	
SSM ‘Canino’	y= 43.600-0.250 x
SSM ‘Mirlo Rojo’	y= 29.600-0.140 x
TIS ‘Canino’	y= 60.000+0.621 x
TIS ‘Mirlo Rojo’	y= 41.420+0.620 x

Table S2. *F*-values obtained in ANOVA analysis for proliferation, shoot length, productivity, leaf surface, fresh weight, and dry weight in two apricot cultivars cultured in two cultivation systems and different AgNPs treatments.

**Source of variation**	**Degree of freedom**	**F-value**	**p>**
Proliferation			
System (S)	1	291.10	0.0001
Cultivar (C)	1	352.68	0.0001
Treatment (T)	4	12.15	0.0001
S x C	1	209.38	0.0001
S x T	4	38.09	0.0001
C x T	4	7.41	0.0001
S x C x T	4	14.23	0.0001
Shoot length			
System (S)	1	14.13	0.0002
Cultivar (C)	1	57.35	0.0001
Treatment (T)	4	1.94	0.1029
S x C	1	0.00	0.9473
S x T	4	1.04	0.3858
C x T	4	3.68	0.0058
S x C x T	4	4.79	0.0009
Productivity			
System (S)	1	365.81	0.0001
Cultivar (C)	1	468.93	0.0001
Treatment (T)	4	10.33	0.0001
S x C	1	243.00	0.0001
S x T	4	31.62	0.0001
C x T	4	5.26	0.0004
S x C x T	4	9.79	0.0001
Leaf surface			
System (S)	1	277.84	0.0001
Cultivar (C)	1	6.14	0.0136
Treatment (T)	4	1.29	0.2744
S x C	1	0.38	0.5373
S x T	4	2.99	0.0185
C x T	4	1.98	0.0959
S x C x T	4	6.44	0.0001
Fresh weight			
System (S)	1	210.84	0.0001
Cultivar (C)	1	15.37	0.0001
Treatment (T)	4	4.82	0.0011
S x C	1	10.66	0.0014
S x T	4	9.78	0.0001
C x T	4	0.05	0.9947
S x C x T	4	0.14	0.9670
Dry weight			
System (S)	1	146.34	0.0001
Cultivar (C)	1	9.27	0.0028
Treatment (T)	4	2.74	0.0310
S x C	1	1.45	0.2308
S x T	4	9.99	0.0001
C x T	4	0.18	0.9503
S x C x T	4	0.13	0.9729

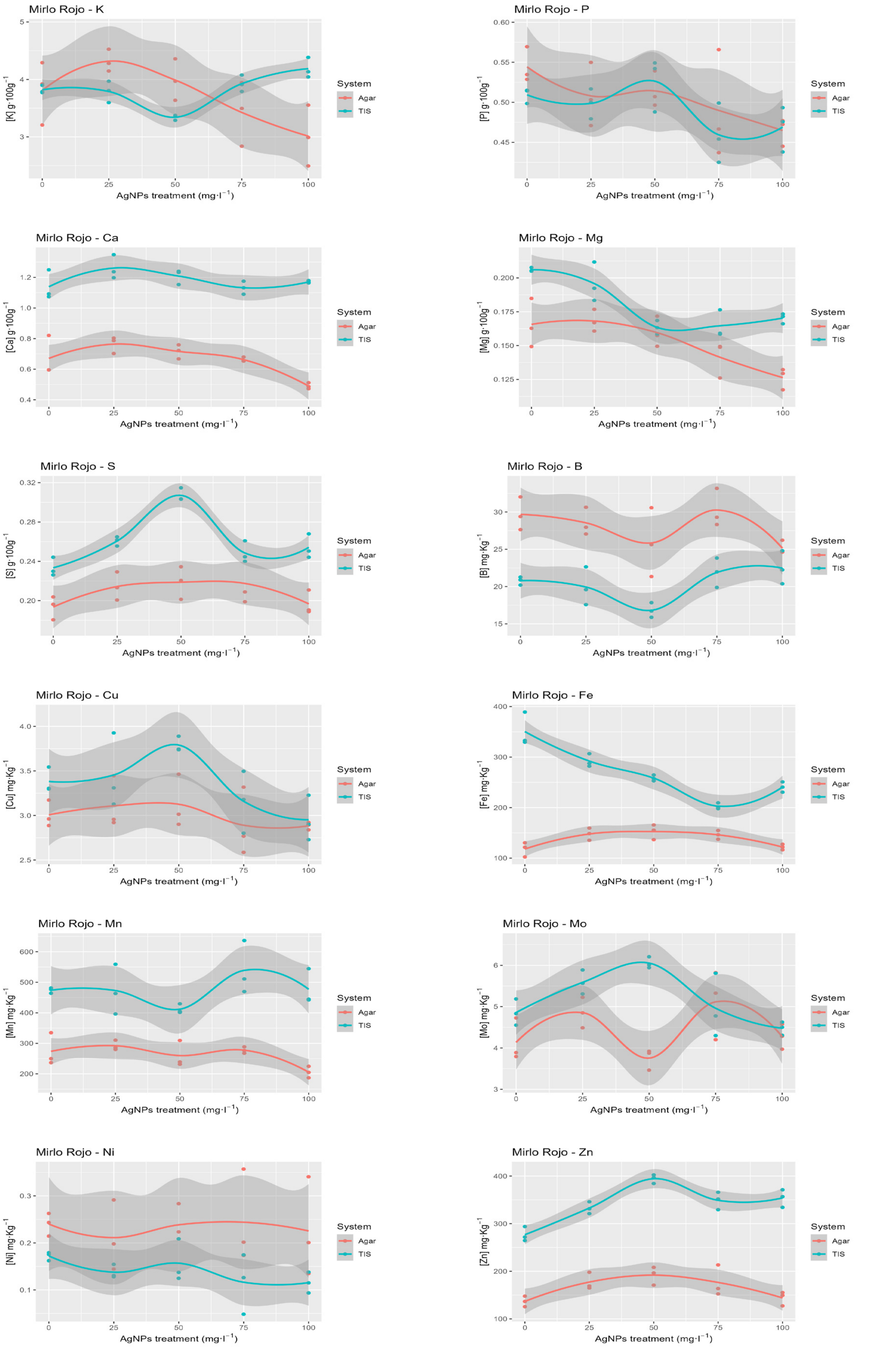

Figure S1. Ions concentration vs. AgNPs concentration for 'Mirlo Rojo'. The line represents the loess regression of the points. Shaded areas represent the 95% confidence interval for the prediction.

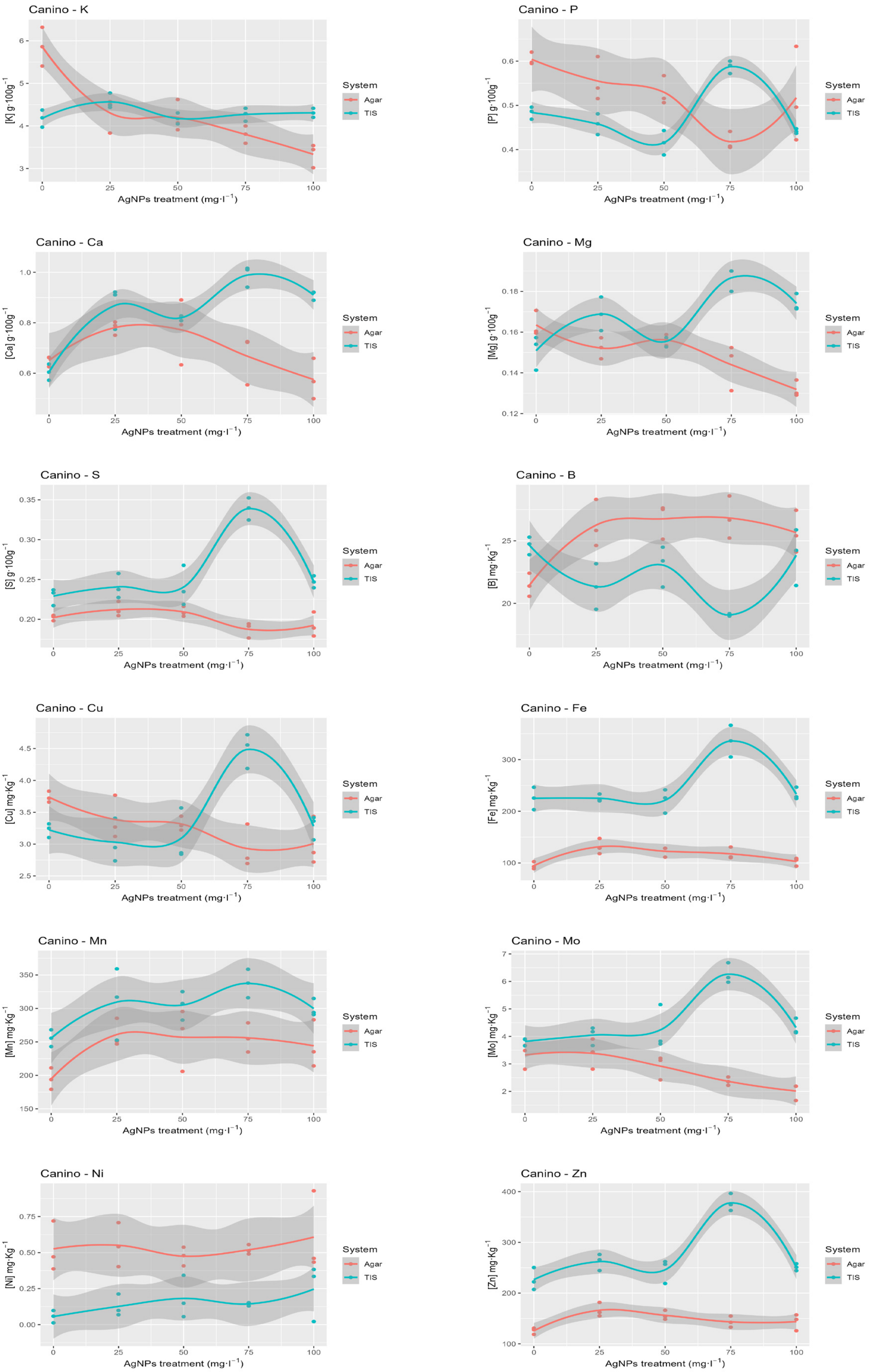

Figure S2. Ions concentration vs. AgNPs concentration for 'Canino'. The line represents the loess regression of the points. Shaded areas represent the 95% confidence interval for the prediction.
